# The N-Terminus of the Floral Arabidopsis TGA Transcription Factor PERIANTHIA Mediates Redox-Sensitive DNA-Binding

**DOI:** 10.1371/journal.pone.0153810

**Published:** 2016-04-29

**Authors:** Nora Gutsche, Sabine Zachgo

**Affiliations:** Botany, University of Osnabrück, Osnabrück, Germany; Ecole Normale Superieure, FRANCE

## Abstract

The Arabidopsis TGA transcription factor (TF) *PERIANTHIA* (*PAN*) regulates the formation of the floral organ primordia as revealed by the *pan* mutant forming an abnormal pentamerous arrangement of the outer three floral whorls. The Arabidopsis TGA bZIP TF family comprises 10 members, of which PAN and TGA9/10 control flower developmental processes and TGA1/2/5/6 participate in stress-responses. For the TGA1 protein it was shown that several cysteines can be redox-dependently modified. TGA proteins interact in the nucleus with land plant-specific glutaredoxins, which may alter their activities posttranslationally. Here, we investigated the DNA-binding of PAN to the *AAGAAT* motif under different redox-conditions. The *AAGAAT* motif is localized in the second intron of the floral homeotic regulator *AGAMOUS* (*AG*), which controls stamen and carpel development as well as floral determinacy. Whereas PAN protein binds to this regulatory *cis*-element under reducing conditions, the interaction is strongly reduced under oxidizing conditions in EMSA studies. The redox-sensitive DNA-binding is mediated via a special PAN N-terminus, which is not present in other Arabidopsis TGA TFs and comprises five cysteines. Two N-terminal PAN cysteines, Cys68 and Cys87, were shown to form a disulfide bridge and Cys340, localized in a C-terminal putative transactivation domain, can be S-glutathionylated. Comparative land plant analyses revealed that the *AAGAAT* motif exists in asterid and rosid plant species. TGA TFs with N-terminal extensions of variable length were identified in all analyzed seed plants. However, a PAN-like N-terminus exists only in the rosids and exclusively Brassicaceae homologs comprise four to five of the PAN N-terminal cysteines. Redox-dependent modifications of TGA cysteines are known to regulate the activity of stress-related TGA TFs. Here, we show that the N-terminal PAN cysteines participate in a redox-dependent control of the PAN interaction with a highly conserved regulatory *AG cis*-element, emphasizing the importance of redox-modifications in the regulation of flower developmental processes.

## Introduction

**Fig 1:**
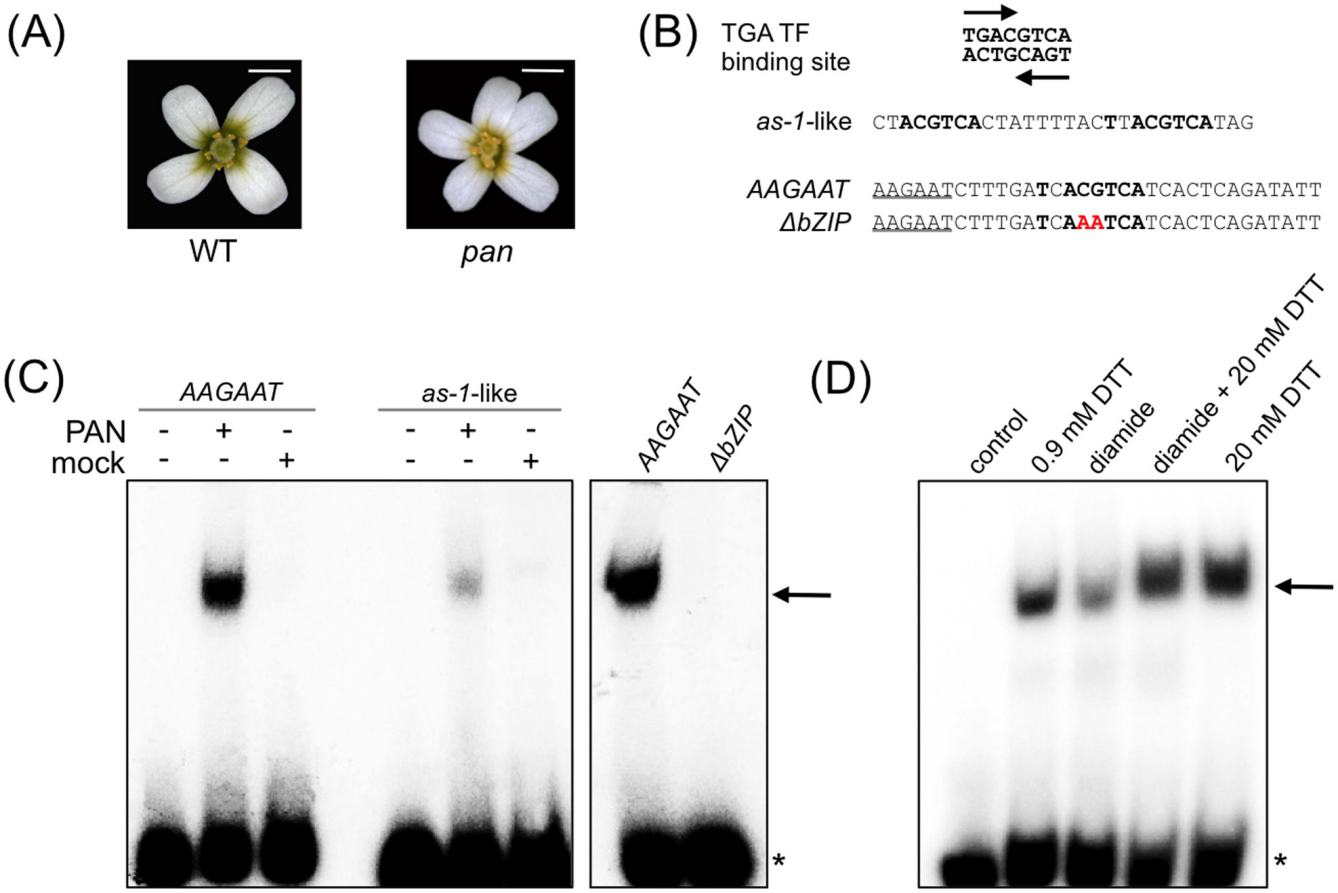
Analysis of redox-dependent PAN DNA-binding. (A) *Arabidopsis* wild-type flowers are composed of four sepals, four petals, six stamens and two fused carpels. Flowers of *pan* mutants form three pentameric whorls with five sepals, five petals, five stamens and two normal carpels. Scale bar 1 mm. (B) Sequence of the palindromic core TGA DNA-binding motif is shown on the top and the three used binding sites comprising the core motif below. The *as-1*-like motif from the Arabidopsis *PR1* promoter comprises two TGA binding sites. The *AAGAAT* motif from the second intron of *AG* encompasses one central TGA binding site with one nucleotide exchange, the name-giving 5’ *AAGAAT* motif is underlined. Mutagenesis of two nucleotides (indicated in red) abolishes binding to the *ΔbZIP* motif. (C) EMSA analysis of the PAN interaction with the *AAGAAT*, *as-1*-like and *ΔbZIP* motifs. Radioactive-labeled DNA probes were incubated with PAN protein or with a mock translation under reducing conditions (0.9 mM DTT). The arrow indicates DNA-protein complex formation and the asterisk marks free DNA probe. D) Analysis of redox-dependent binding of PAN to the *AAGAAT* motif. For comparison of reducing and oxidizing conditions, PAN protein was incubated prior to DNA-binding with 0.9 mM or 20 mM DTT and with 2 mM diamide, respectively. Reversibility of the redox-dependent DNA-binding was analyzed by the addition of 20 mM DTT after a 2 mM diamide treatment. The control reaction was conducted without protein addition.

Angiosperm flowers are commonly composed of sepals, petals, stamens and carpels and variations in the number and shape of these organs contribute to generate the enormous flower diversity. The capacity to reproduce sexually is a crucial step in the plant life cycle and flower organ development is controlled by a complex regulatory network involving the transcriptional as well as posttranscriptional regulation of key floral transcription factors (TFs). The bZIP TF *PERIANTHIA* (*PAN*) regulates the initiation of floral organ primordia in the outer three floral whorls and belongs to the *TGACG* (TGA) motif-binding family. Loss-of-function *pan* plants form pentamerous flowers with five sepals, five petals and five stamens, compared to the development of four sepals, four petals and six stamens in wild-type flowers (Fig 1A) [1]. The TGA TF family of Arabidopsis consists of 10 members known to participate in flower developmental and stress response processes [2]. They execute their activity by binding to *cis*-regulatory elements comprising the name-giving TGACG core recognition sequence. A well-characterized TGA TF binding element is the *activation sequence-1* (*as-1*) motif, first described in bacterial and viral promoters [3]. Variations of this motif, named *as-1-like* motifs, are present in promoter regions of various stress-related plant genes, such as *PATHOGENESIS-RELATED GENE1 (PR1)* or *GLUTATHIONE-S-TRANSFERASE* (*GST*) [4–8]. Using yeast one-hybrid and X-Chip approaches, PAN was found to bind to the *AAGAAT* motif in the second intron of the key flower development regulator *AGAMOUS* (*AG*) [9, 10]. The 33 bp long *AAGAAT* response element includes a central TGACG core recognition sequence, the minimal requirement for TGA TF binding, which is flanked by a pair of conserved GARP-binding elements, where the 5’ end starts with the name-giving AAGAAT sequence [9] (Fig 1B). The motif had originally been identified by a combination of phylogenetic footprinting and shadowing approaches in the Brassicaceae and other eudicots [11] and has later been shown to exist also in a monocot species [12]. The importance of the *AAGAAT* motif for the *AG* expression regulation has been demonstrated by a strongly reduced *AG* expression in a GUS-reporter line lacking the *AAGAAT* response element [9]. In Arabidopsis, *AG* exerts the class C homeotic function of the floral ABC model and acts in the third whorl together with class B genes to control stamen formation and governs carpel organogenesis in the fourth whorl [13]. Additionally, *AG* controls flower determinacy, as the central fourth whorl is replaced by an indeterminate flower composed of sepals and petals in *ag* mutants. The different roles of the class C function are realized by a precise temporal and spatial as well as quantitative *AG* expression regulation [12, 14]. The second *AG* intron also comprises binding sites for other key floral regulators such as *WUSCHEL* and *LEAFY* and serves as a crucial hub integrating different signals to mediate a complex expression regulation [9, 11].

It has been shown that a nuclear interaction of PAN with ROXY1, a plant-specific glutaredoxin (GRX) is crucial to govern petal development [15]. GRXs are glutathione-dependent oxidoreductases that participate in diverse redox-regulated processes. The PAN Cys340 has been shown to be crucial for the in vivo activity of PAN and represents a potential target site for posttranslational modifications [15]. Whereas the precise biochemical activity of the plant-specific GRXs remains elusive, ubiquitously occurring GRXs are known to modulate target protein activities by S-deglutathionylation reactions or by the reduction of protein disulfide bonds [16]. TGA9 and TGA10 also interact with ROXY1 and its closest homolog ROXY2 in the nucleus and redundantly regulate male germ line formation [17]. This raises the question of whether redox-processes might also be involved in a posttranslational regulation of nuclear PAN activity during flower development as it was previously shown for TGA1, which is involved in mediating basal resistance by an salicylic acid (SA)-dependent pathway [16, 18]. Two TGA1 cysteine residues, Cys260 and Cys266, form a disulfide bridge under oxidizing conditions, which impedes the interaction with its co-factor NON EXPRESSOR OF PATHOGENESIS RELATED GENES1 (NPR1). Under reducing conditions, no disulfide bridge is formed and this enables the interaction with NPR1, which enhances the binding to the stress-related *as-1-*like element [19].

In this study, we investigated whether PAN interacts redox-dependently with the *AAGAAT* and *as-1-*like motif. PAN strongly interacts only under reducing conditions with these regulatory *cis*-elements and the redox-dependent DNA-binding is mediated by the combinatorial activity of the five N-terminal PAN cysteines. Modifications of three PAN cysteine residues were identified in vitro and S-glutathionylation of Cys340 localized in a putative transactivation domain indicates that additional redox-dependent mechanisms can modify the PAN activity posttranslationally. Finally, comparative evolutionary analyses revealed that the PAN-like N-terminus exists only in Brassicaceae PAN homologs and evolved after the origin of the *AAGAAT* motif, likely coinciding with the successful radiation of this family.

## Material and Methods

### Plant material, growth conditions

Seeds of *Arabidopsis thaliana* Col-0 and transgenic plants were grown on soil at 20°C for four weeks under short-day conditions (8 h light) and then transferred to long-day conditions (16 h light) at 55% relative humidity. Transgenic T_1_ plants were selected by spraying 0.2% BASTA^®^ (BAYER) (v/v) solution onto the seedlings.

### Isolation and cloning of TGA TF family members

Total RNA was isolated with the RNeasy Plant Mini kit (Qiagen) from *Arabidopsis thaliana* WT leaves. 2 μg of RNA was applied for cDNA synthesis using the SuperScript^®^ II Reverse Transcriptase (Invitrogen) and an oligo(dT) primer (Roche). To obtain the full-length coding sequence of *PAN*, *TGA1*, *TGA2*, *TGA3* and *TGA10*, amplifications using gene-specific primers with restriction sites were performed. Fragments were cloned into the pTnT^™^ vector (Promega) and sequence accuracy verified. All primers with restriction sites indicated as well as the accession numbers are listed in S1A and S1B Table.

### Site-directed mutagenesis

Cysteine-to-serine substitution mutants were created by a PCR-based mutagenesis approach utilizing previously generated single PAN cysteine-to-serine mutant templates together with paired mutagenic oligomers as described in [15]. The obtained novel mutated PAN versions (*PANC68SC87S*, *PANC27SC114SC154SC340S*, *PANC27SC68S*, *PANC27SC68SC87S*, *PANC27SC68SC87SC114S*, *PANC27SC68SC87SC114SC154S*, *PANC27SC68SC87SC114SC154SC340S*) and the six PAN single cysteine-to-serine mutants were cloned into the pTnT^™^ vector using *Kpn*I and *Xba*I restriction sites. In addition, a N-terminal deletion variant of *PAN* (*PANΔN*), lacking amino acids 1–166 and harboring an artificial start methionine codon, was amplified and inserted into the pTnT^™^ vector.

### Complementation experiments

For in planta complementation analysis of the *pan* mutant, full-length cds of *PAN*, *MBP-PAN*, *PANC68SC87S*, *PANC27SC68SC87SC114SC154S* and *PANΔN* were cloned into the pBAR-35S vector using *Xma*I and *Xba*I restriction sites. All constructs were introduced into *Agrobacterium tumefaciens* strain GV3101 (pMP90RG) and transformations of *pan* (SALK_057190) plants were conducted as described in [15]. At least 200 flowers from 20 transgenic T_1_ plants were examined for complementation of the petal phenotype.

### Electrophoretic mobility shift assay

Gel retardation assays were done with in vitro generated proteins using the TnT^™^ SP6-Coupled Reticulocyte Lysate system (Promega) according to the manufactures instructions and ^35^S-labeled methionine was added to enable protein synthesis and detection (S1 Fig). For DNA probe preparation, purchased oligonucleotides (S1A Table) were labeled with γ-P^32^ at the 5’ ends using a T4 polynucleotide kinase (Thermo Scientific) according to the manufactures instructions. EMSAs were performed as described in [20] with a reaction mix with or without DTT (redox EMSA). For redox EMSAs, deviating from [20], either DTT (0.9 mM, 20 mM) (Roth) or 2 mM diamide (Sigma-Aldrich) was added. Probes were incubated for 30 min in the dark prior to DNA application. The potential re-reduction of previously diamide-treated reaction mixtures was achieved by addition of 20 mM DTT after the first incubation step followed by a second 30 min incubation step in the dark prior to DNA probe addition. Complex formation was detected by autoradiography after overnight exposure using a Storage Phosphor Screen (GE Healthcare) and visualized with a Storm^™^820^®^ PhosphorImager (Molecular Dynamics, Amersham). All EMSA experiments were repeated at least three times with different in vitro translation products.

### Cloning, expression, and purification of recombinant protein

Full-length cds of *PAN* and *PANC27SC68SC87SC114SC154SC340S* (*PAN6xCysmut*, ~50 kDa) were amplified and cloned into the pMAL-c5X vector (NEB) in frame with the maltose binding protein (MBP, ~43 kDa) sequence using *Xmn*I and *Nco*I restriction sites. Verified plasmids were transformed into the BL21 (DE3) *Escherichia coli* strain (Novagen). Recombinant protein was produced and purified according to the manufactures protocol. Reducing SDS-PAGE analysis was performed to ensure successful purification. Protein concentrations were determined by conducting a Bradford assay using the Coomassie Plus^™^ Protein Assay reagent (Thermo Scientific).

### Non-reducing SDS-PAGE analysis

MBP-PAN and MBP-PAN6xCysmut protein samples (10 μg) were treated with either 20 mM DTT, 2 mM diamide or were first incubated with 2 mM diamide for 30 min followed by an additional 30 min incubation step with 20 mM DTT for 30 min. Free cysteines were blocked with 20 mM iodacetamide and an 1 h incubation in the dark. Probes were precipitated with trichloroacetic acid, pellets were washed twice with ice-cold 100% actone and resuspended in 1x Laemmli-buffer without any reducing agents and boiled for 5 min at 95°C. Protein samples were loaded on a 4–20% Mini-Protean^®^ TGX^™^ precast gel (Bio-Rad) and electrophoresis was performed in 1x SDS-PAGE running buffer. Staining with PageBlue^™^ protein staining solution (Thermo Scientific) was performed for visualization of the protein.

### Identification of cysteine modifications via tandem mass spectrometry

Purified recombinant MBP-PAN protein (30 μg) was incubated with 5 mM GSH (Sigma-Aldrich) and 5 mM H_2_O_2_ (Roth) or with 10 mM GSSG (Roth) for 30 min in the dark, alkylated and separated on a non-reducing SDS-PAGE gel. Interesting proteins bands were cut out of the gel and incubated in destaining solution (100 mM NH_4_HCO_3_, pH 8.5, 30% acetonitrile). Bands were washed in LC-MS CHROMASOLV^®^ water (Fluka), incubated in acetonitrile and an in-gel digestion using endoproteinase AspN (Roche) was performed according to the manufactures instruction. Peptides were analyzed using a two-step LC-ESI MS/MS system. First, peptides were separated in a nanoHPLC (Ultimate^®^ 3000 HPLC, Dionex) and then ionized using electrospray ionization (CapticeSpray, Bruker). The ionized peptides were further analyzed according to their mass/charge values using an ion trap (amaZon speed ETD, Bruker). Second, selected peptides were additionally analyzed by fragmentation analysis using the electron transfer dissociation (ETD) technique. This method leads to an induced disulfide bond cleavage supporting the identification of posttranslational cysteine modifications related to disulfide bond formation [21, 22]. Evaluation of data was done with a Mascot-based program for analyzing disulfide bond formation provided by the MS facility of the University of Osnabrück. The program was used to compare the masses of the predicted, unmodified cysteine-containing PAN peptides with the masses obtained by the two MS analyses.

### In silico analysis

Sequence data from PAN and *AG* homologs were obtained from databases provided by PLAZA (Dicot 3.0 and Monocot 3.0) [23], phytozome v11 [24], the ancestral angiosperm genome project (AAGP; www.ancangio.uga.edu), congenie.org (congenie.org) [25], Brassica Database (BRAD; brassicadb.org), Comparative Genomics (CoGeBlast; genomevolution.org) [26] and NCBI. Blastp and blastn searches were performed with NCBIblast and blast tools provided from the different sources with standard parameters using the PAN full-length protein sequence, the *AAGAAT* motif as well as the *AG* sequence. Comparison of the N-termini and the cysteine residue presence was done based on protein alignments shown in S3 Fig using the CLUSTALW algorithm (MacVector, version 12.0.2). For *Liriodendron tulipifera* (magnoliids) definition of a start codon was not possible and genome data were not available. To identify to presence of the *AAGAAT* motif in genomic sequences of *AG* homologs, genomic sequences were aligned (S2 Fig) and the respective sources and accession numbers are listed in S1C Table. It was discriminated between partial *AAGAAT* motifs and *AAGAAT* motifs. Both contain the *AAGAAT* site, where the first A of the AAGAAT sequence can be replaced by a C or T. A partial motif does not comprise a TGA core binding sequence and more than 15 nucleotide deviations occur in the 33 bp sequence compared to a maximum of seven in the *AAGAAT* motif. A cladogram based on a phylogenetic tree from [27] was used compare the evolutionary relationships of the PAN N-termini, the N-terminal cysteine residues and the *AAGAAT* motif.

## Results and Discussion

### The TGA transcription factor PERIANTHIA binds in a redox-dependent manner to DNA motifs

Plant-specific TGA TFs exert their activity by binding to characteristic *cis*-elements in regulatory regions of target genes comprising the TGACG core sequence [3]. Electrophoretic mobility shift assays (EMSAs) were conducted to analyze the ability of PAN to bind to two known TGA TF response elements comprising the *TGACG* binding site. To start, we compared the in vitro binding capacity of PAN to the *AAGAAT* motif from the second intron of the floral regulator *AG* and the stress-related *as-1*-like motif from the *A*. *thaliana PR1* promoter region (Fig 1B) [4, 9, 10, 28]. The *as-1*-like motif contains two and the *AAGAAT* motif one *TGACG* core recognition site in a reverse-complement orientation (Fig 1B). In vitro produced PAN protein was incubated with the radioactively-labeled DNA motifs. Additionally, a mutagenized *AAGAAT* variant, the *ΔbZIP* motif was used, which comprises a two-nucleotide exchange in the TGACG core sequence shown to abrogate the PAN-DNA interaction in yeast one-hybrid studies [9] (Fig 1B). PAN binding to the *AAGAAT* and *as-1*-like motif is demonstrated by the appearance of shifted bands in the EMSA analysis, representing the respective PAN-DNA complexes (Fig 1C; arrow), whereas the PAN-DNA interaction is lost using the *ΔbZIP* motif (Fig 1C). The EMSA data show that PAN binds in vitro to developmental as well as stress-related *cis*-regulatory elements.

PAN exhibited a stronger binding affinity for the *AAGAAT* element, even though the *as-1*-like motif contains two potential TGA TF binding sites (Fig 1B and 1C). The *as-1*-like and *AAGAAT* motifs deviate from the perfect TGF TF recognition site TGACGTCA by comprising one nucleotide exchange (Fig 1B). Positive as well as negative effects of single nucleotide variations in response elements on the interaction with TFs have been described. The DNA-binding affinity of the human HEPATOCYTE NUCLEAR FACTOR-1α is increased if a substituted motif variant comprising a T to G exchange is analyzed and a single nucleotide binding site alteration affects the DNA-interaction affinity of the JUN-FOS TF heterodimers negatively [29, 30]. The difference in PAN binding affinities indicates that the presence of an AAGAAT sequence preceding a single TGA core motif enhances the interaction capacity.

For TGA1, posttranslational cysteine modifications of Cys260 and Cys266 were shown to affect its DNA-binding capacity [19]. Given that Cys340 was shown to be crucial for mediating an in vivo PAN function [15] and that it is equivalent to the TGA1 Cys260, we next investigated whether PAN binds in a redox-dependent manner to the *AAGAAT* motif. A redox-sensitive DNA-binding of the PAN protein was detected by conducting redox EMSAs where the PAN protein was either incubated with the reducing agent DTT (20 mM) or with the oxidant diamide (2 mM) for 30 min prior to the incubation with the *AAGAAT* motif. PAN bound to the *AAGAAT* motif under reducing conditions, whereas incubation with diamide led to a strongly decreased PAN-DNA interaction (Fig 1D). Next, the reversibility of the redox-dependent binding was analyzed. The PAN reaction mix was first oxidized with 2 mM diamide followed by incubation with a 10-fold higher concentration of DTT (20 mM) before the DNA probe was added. Thereby, the interaction of the PAN protein with the *AAGAAT* motif could be restored. The reversibility of the PAN-DNA-binding suggests that redox-dependent modifications can occur, which enable a specific and rapid change of the PAN protein activity. Examples for redox-regulated TFs participating in stress responses are Rap2.4, which controls the expression of a 2-Cys peroxiredoxin and bZIP16, a TF participating in light-stress induced responses [31, 32]. The redox-sensitive homeodomain-zipper TF HAHR1 has been suggested to regulate the development of epidermal cells and the bHLH TF TCP15 is likely involved in both, developmental- and stress-related pathways [33–35]. Together, these data show that redox-dependent DNA-binding represents a mode of posttranslational protein activity regulation for different TF classes including the TGA TFs TGA1 and PAN, which participate in stress-related and developmental processes, respectively.

### Binding of TGA family members to the *AAGAAT* motif differs in response to altered redox-conditions

**Fig 2:**
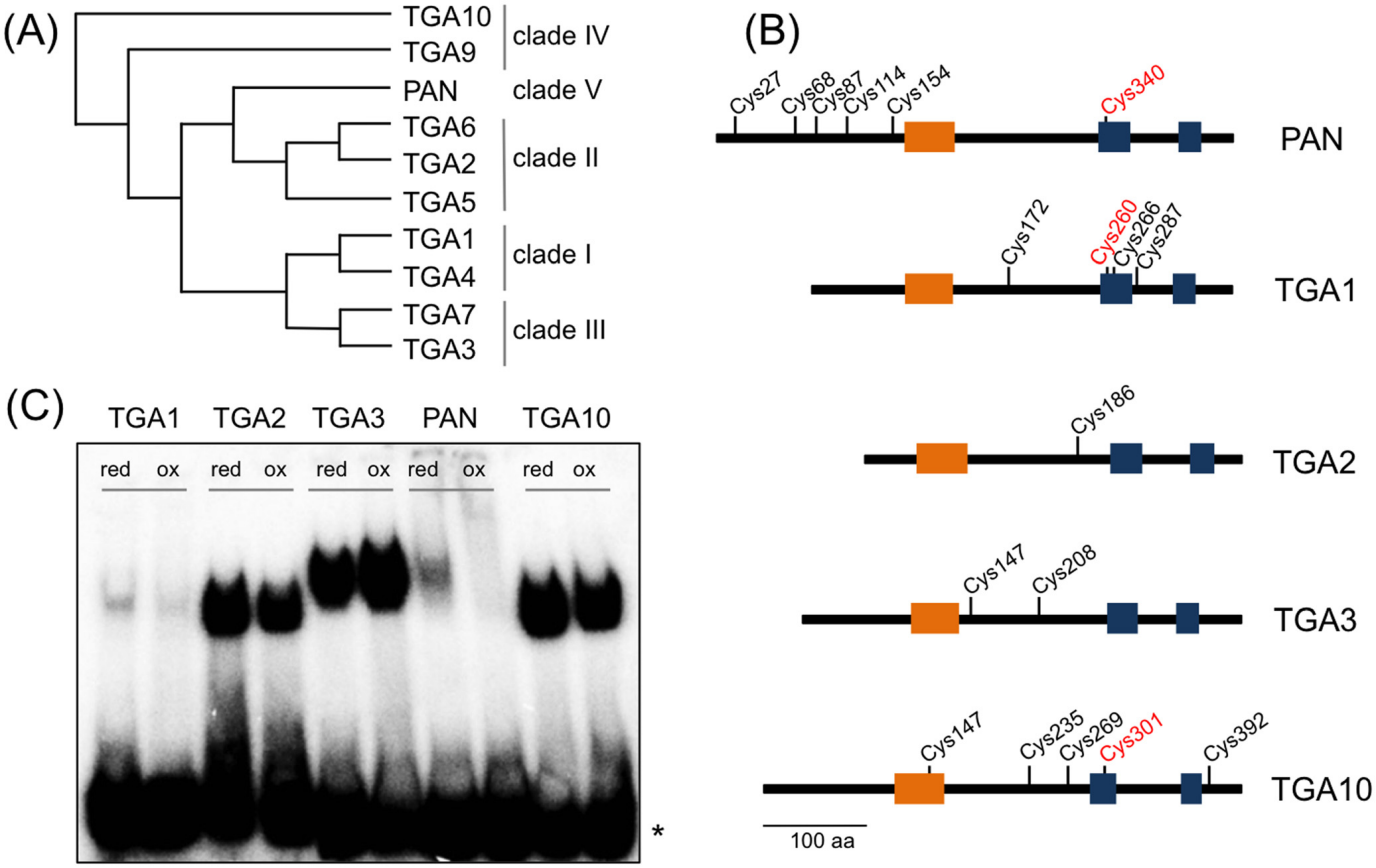
Redox-dependent binding of selected Arabidopsis TGA TFs to the *AAGAAT* motif. (A) Cladogram of Arabidopsis TGA TFs indicating clade memberships. (B) Schematic representation of selected TGA TF family members and respective cysteine residue positions. Orange boxes mark the bZIP domain and blue boxes two glutamine-rich regions. Cys340, Cys260 and Cys301 (red) are localized in a conserved position in the first glutamine-rich region. (C) Gel retardation analysis of selected TGA proteins incubated with the *AAGAAT* probe under reducing (0.9 mM DTT; red) or oxidizing (2 mM diamide; ox) conditions. Asterisk marks unbound *AAGAAT* probe.

The Arabidopsis TGA TF family comprises five clades (Fig 2A) [2]. Clade I-III members are mainly involved in stress-defense reactions, whereas clade IV and V TGA TFs participate in flower development [1, 15, 17, 36–42]. To further investigate a redox-dependent DNA-binding of the TGA TF family, TGA1, TGA2, TGA3 and TGA10 were selected as representative members of the five clades and analyzed in comparison to PAN by redox EMSAs (Fig 2B and 2C). TGA1 acts in basal resistance processes against virulent *Pseudomonas syringae* strains via an SA-dependent interaction with NPR1 and contributes to additional defense mechanisms [39, 43]. TGA2 and other clade II members exert redundant functions in the SA-dependent systemic required resistance and during the activation of jasmonic acid (JA)- and ethylene (ET)-dependent defense mechanisms [40, 44]. TGA3, like TGA1, mediates a basal resistance and additionally conducts a function in cadmium uptake and transport [36, 37]. Whereas PAN acts during early flower development, TGA9 and TGA10 redundantly control later flower developmental processes, namely microsporogenesis and tapetum formation [17]. The respective TGA1, TGA2, TGA3, TGA10 and PAN proteins were produced in vitro and redox EMSAs were performed using the *AAGAAT* probe. All TGA TFs bound to the *AAGAAT* motif under reducing conditions (Fig 2C) and alteration of redox-conditions revealed weaker effects on the DNA-binding capacities of TGA1, TGA2, TGA3 and TGA10 in comparison to PAN (Figs 1D and 2C). Sequence comparisons were conducted with the selected TGA proteins to reveal conserved and non-conserved cysteine residues (Fig 2B). The PAN protein comprises six cysteines representing putative posttranslational modification sites [15]. Five cysteine residues are localized in the N-terminal sequence extension, and these are unique for PAN as they are absent in all other TGA TFs. The sixth, functionally important Cys340 is located in a position equivalent to the Cys260 in TGA1, for which it is known that it can form a disulfide bridge under oxidizing conditions with the Cys266 of TGA1. PAN does not possess a Cys266 equivalent and these observations raise the question about the possible types of the PAN cysteine modifications.

### Posttranslational modifications of PAN cysteines

**Fig 3:**
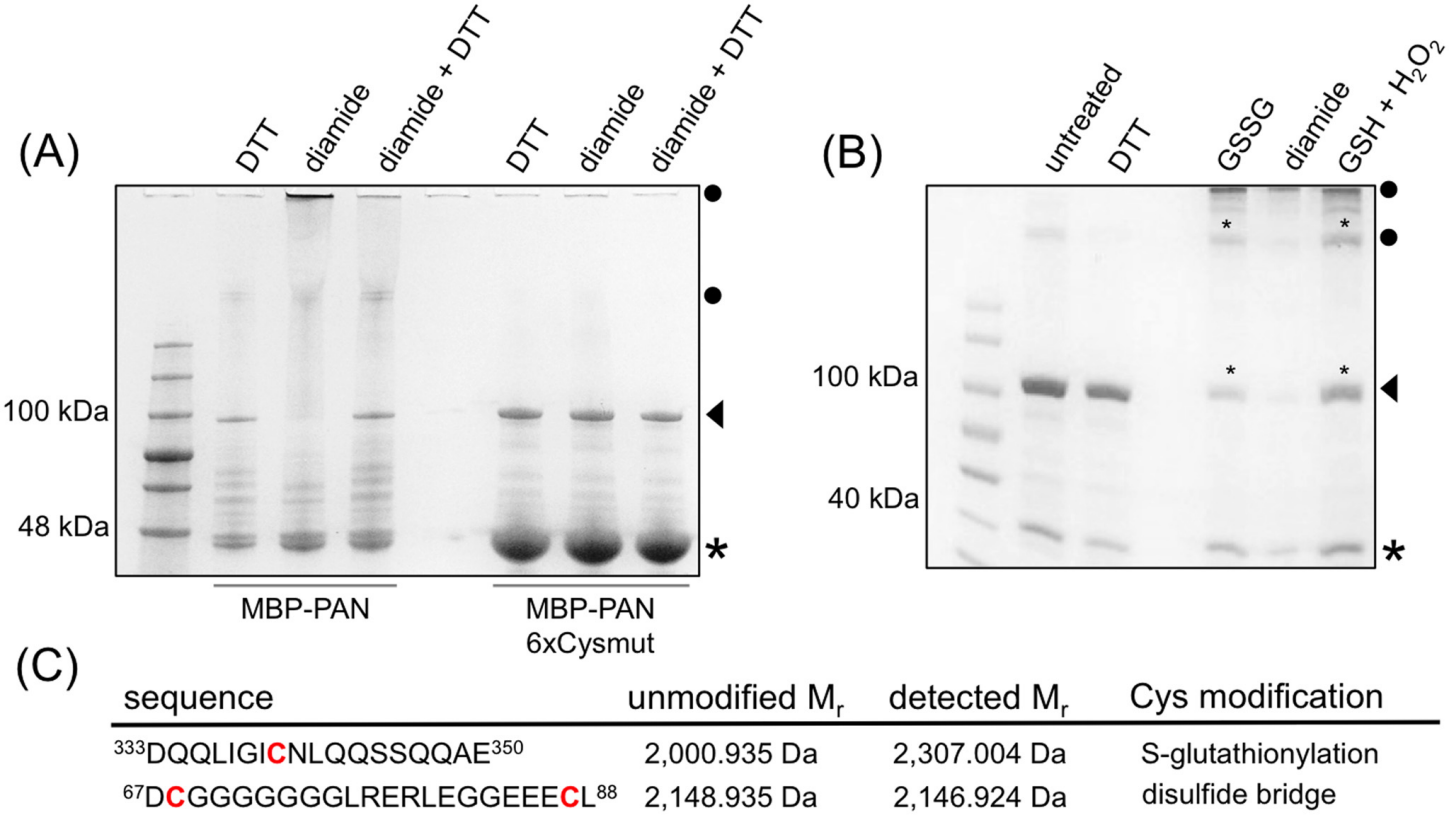
Analysis of posttranslational PAN modifications. (A) Non-reducing SDS-PAGE analysis of MBP-PAN and MBP-PAN6xCysmut proteins. Proteins were incubated with 20 mM DTT, 2 mM diamide or with 2 mM diamide followed by 20 mM DTT. Triangles indicate MBP-PAN (~90 kDa) and MBP-PAN6xCysmut (~90 kDa) proteins and the asterisk marks MBP (~43 kDa). Dimerized and oligomerized fusion proteins are labeled with full circles. (B) Non-reducing SDS-PAGE analysis for identification of posttranslational cysteine modifications. MBP-PAN protein was incubated under different conditions (20 mM DTT, 2 mM diamide, 10 mM GSSG and 5 mM GSH/5 mM H_2_O_2_) and interesting bands, marked with an asterisk, were further processed for MS analysis. (C) Posttranslational PAN cysteine modifications as determined by the difference between predicted, unmodified and detected MS mass values.

Therefore, we next aimed to identify the PAN cysteines and their posttranslational modifications, which potentially mediate the redox-sensitive PAN DNA-binding. Full-length PAN protein and a mutant version, where all six cysteines were replaced by serines (PAN6xCysmut), were expressed with an N-terminal maltose binding protein (MBP) fusion to facilitate soluble protein production and increase protein stability. The MBP and linker region do not contain any cysteine residues, which could affect the PAN protein redox-analysis. The effect of reducing and oxidizing agents on MBP-PAN and MBP-PAN6xCysmut proteins was analyzed by non-reducing SDS-PAGE experiments. Under reducing conditions (20 mM DTT), a band of about 90 kDa represents the MBP-PAN fusion protein (Fig 3A; triangle). In addition, bands displaying the MBP alone (~43 kDa; small asterisk) were detected as well as bands migrating between the MBP-PAN and the MBP bands, which likely represent degradation products. Incubation of MBP-PAN with diamide resulted in additional high molecular weight bands indicating an oligomerization of the fusion protein (Fig 3A; filled circle), which showed a reversible response to changed redox buffer conditions. Contrarily, after diamide treatment the MBP-PAN6xCysmut fusion protein behaved like the reduced MBP-PAN protein and did not oligomerize (Fig 3A). To exclude a negative effect of the MBP-tag on the PAN function, complementation experiments of the *pan* knockout plants were performed as described in Li et al. (2009) [15]. Flower phenotypes of transgenic *pan* plants constitutively expressing *MBP-PAN* under the control of the *CaMV 35S* promoter were analyzed (n = 200 flowers, 20 T_1_ lines). T_1_ plants formed WT-like flowers and the complemented flower phenotype demonstrated the in planta functionality of MBP-PAN fusion proteins. Several different posttranslational regulation mechanisms regarding cysteine modifications have been described for plant TFs, such as the formation of intra- or intermolecular disulfide bridges, S-glutathionylation and S-nitrosylation, as well as the oxidation of cysteine sulfhydryl groups [34, 45]. MBP-PAN was treated with redox-active substances to determine posttranslational PAN cysteine modifications by mass spectrometric analysis (MS). Recombinant protein was incubated in vitro either with 10 mM GSSG or with 5 mM GSH and 5 mM H_2_O_2_ and then separated on a non-reducing SDS-PAGE. Incubation with the modifying substances resulted in the appearance of several bands representing monomeric and higher oligomeric MBP-PAN variants, which is in agreement with the results obtained for the diamide-treated probes (Fig 3B). Four bands with potentially modified MBP-PAN proteins were cut out and an in-gel digestion followed by tandem mass spectrometric analysis was performed (Fig 3B; small asterisks). Two different PAN cysteine modifications were detected, independently of its oligomeric state after incubation with GSSG or GSH/H_2_O_2_ (Fig 3). Based on a mass difference of 306 Da representing a glutathione moiety, S-glutathionylation was identified for Cys340 (Fig 3C) and further confirmed by the ETD technique [21, 46, 47], where a dissociation of the glutathione was detected due to disulfide bond cleavage. S-glutathionylation of specific cysteine residues has been shown to alter TF activity at different regulatory levels. Frequently, S-glutathionylation is associated with a reduced DNA-binding capacity as described for the human TFs PAX-8, c-JUN and the p50 subunit of the NUCLEAR FACTOR κB (NFκB) [48–51]. For another NFκB subunit, p65, S-glutathionylation of specific cysteines results in inhibition of the nuclear translocation [52]. Additionally, S-glutathionylation can also have a protective effect and prevent the irreversible oxidation of cysteine sulfhydryl groups [53]. TGA1 was found to be S-glutathionylated and S-nitrosylated after treatment with S-nitrosoglutathione, potentially protecting TGA1 cysteines from irreversible oxidative modifications and enhancing in vitro the DNA-binding of TGA1 in the presence of NPR1 [45]. For PAN, functional complementation data with single cysteine mutants suggested that the detected S-glutathionylation of Cys340 is crucial for PAN to exert its activity during flower development [15]. The second identified posttranslational modification is the formation of a disulfide bridge between Cys68 and Cys87, both located in the PAN N-terminus (Fig 3C). An intramolecular disulfide bridge formation was detected for the Cys68Cys87-containing peptide that resulted in loss of two hydrogen atoms in the MS analysis (Fig 3C), which was supported by ETD analysis. Disulfide bridge formation can regulate the activity of TFs by changing the structure of TF dimers or by affecting the oligomerization state [32, 54–56]. Recently, the impact of intermolecular disulfide bridge formation has been demonstrated for the plant-specific TCP TFs TCP15 and TCP16. Under oxidizing conditions, these TFs form an intermolecular disulfide bridge between the two respective monomers, thereby omitting an interaction with DNA response elements [34]. A disulfide bridge formation between Cys260 and Cys266 from TGA1 negatively affects the interaction capacity with the co-factor NPR1. PAN cysteine modifications of Cys340 and Cys68/Cys87 occurred in vitro under high GSSG or H_2_O_2_ and GSH concentrations and therefore support a regulation of the PAN activity by posttranslational cysteine modifications. No peptides comprising Cys27, Cys114 and Cys154 from the PAN N-terminus could be detected with this approach. Therefore, additional modifications of these cysteines may occur and contribute to the posttranslational PAN regulation.

### The PAN N-terminus mediates redox-sensitive DNA-binding

**Fig 4:**
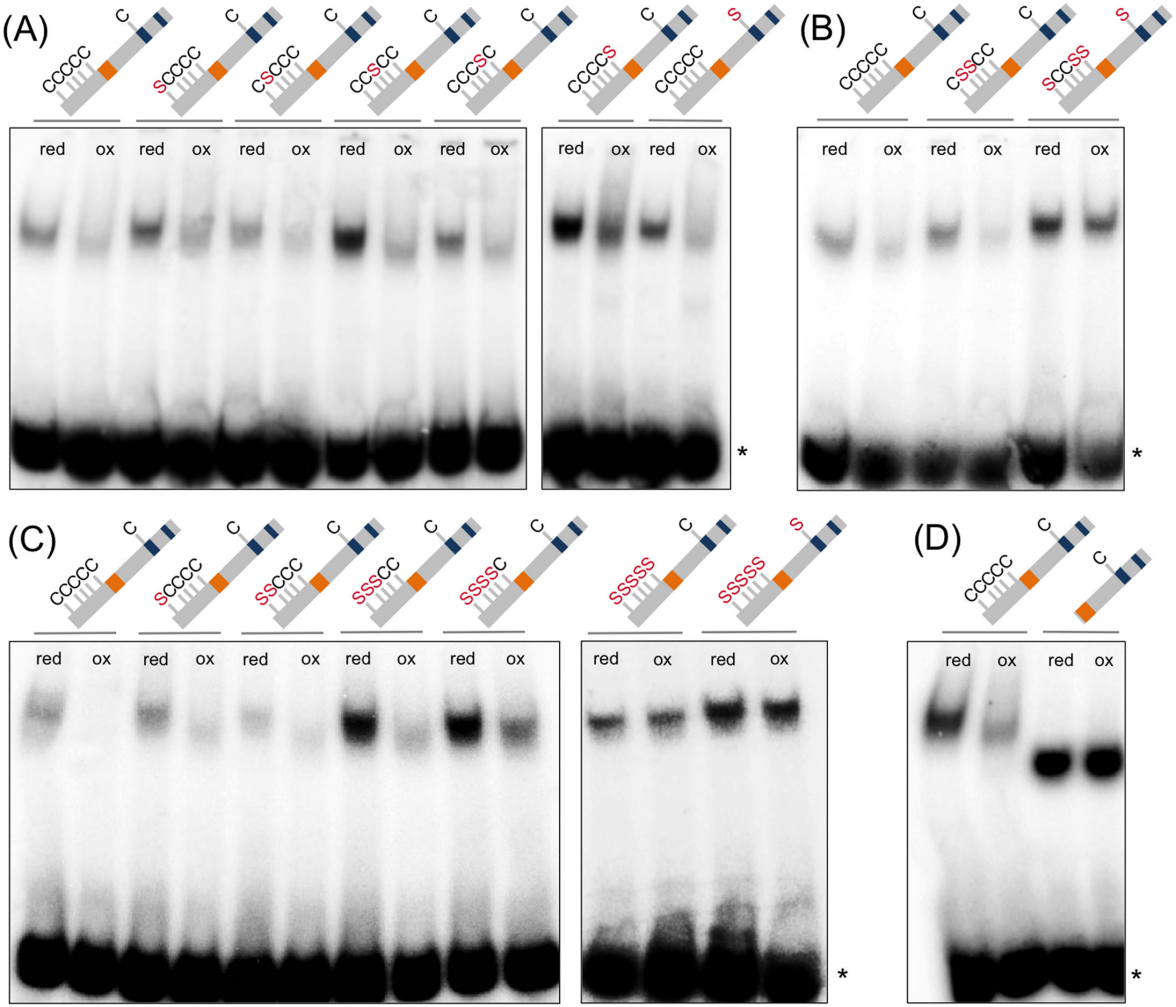
Redox-dependent DNA-binding of PAN is mediated via the N-terminal cysteines. Redox EMSA analyses to investigate the influence of single (A) and multiple Cys-to-Ser substitutions (B and C) and the effect of a N-terminal PAN truncation (D). Schematic illustrations of the PAN variants indicate the bZIP domain in orange and the glutamine-rich regions in blue boxes. Cys-to-Ser substitutions are labeled in red. PAN proteins were incubated in 0.9 mM DTT (red) or 2 mM diamide (ox) prior to addition of the *AAGAAT* motif. Asterisks mark unbound free probe.

The contribution of the six PAN cysteines to mediate a redox-sensitive DNA-binding was investigated using different individual and combined PAN cysteine-to-serine substitutions. Due to lack of a sulfhydryl group in the serine, substitutions of cysteine by serine structurally mock a reduced cysteine state and thus abrogate posttranslational redox-modifications requiring the oxidation of the cysteine sulfhydryl group. Six PAN cysteine single substitution mutants (PANC27S, PANC68S, PANC87S, PANC114S, PANC154S, PANC340S) were generated. Under oxidizing conditions, all single cysteine-to-serine PAN substitution proteins maintained a decreased *AAGAAT* motif binding affinity (Fig 4A), including also Cys340, which can be S-glutathionylated. Although Cys340 is found in a conserved position with Cys260 in TGA1, our data indicate that it is neither involved in disulfide bridge formation nor contributes to the redox-dependent DNA-binding. Cys340 regulates the PAN activity likely positively [15] and S-glutathionylation may protect Cys340 from an irreversible and thus deleterious overoxidation. Single substitutions of the two residues Cys68 and Cys87 for which MS analyses showed a disulfide bridge formation after GSSG and H_2_O_2_/GSH treatment also did not affect redox-sensitive DNA-binding. The influence of Cys68 and Cys87 was analyzed by testing the DNA-binding capacity of PAN mutant proteins where both cysteines were substituted (PANC68SC87S) and by a quadruple mutant variant where all cysteines were exchanged, except for Cys68 and Cys87 (PANC27SC114SC154SC340S). The PANC68SC87S double mutant still bound in a redox-sensitive manner to the *AAGAAT* motif (Fig 4B). However, in the reverse situation, where the quadruple N-terminal PAN mutant harbored only the functional Cys68 and Cys87 residues, a weak decrease was observed in the interaction with the *AAGAAT* motif after diamide treatment, indicating that other cysteines contribute to the redox-sensitive DNA-binding.

To dissect the impact of the PAN N-terminal cysteines on the redox-sensitive DNA-binding, serially increased PAN cysteine-to-serine substitution mutants (PANC27S, PANC27SC68S, PANC27SC68SC87S, PANC27SC68SC87SC114S, PANC27SC68SC87SC114SC154S, PANC27SC68SC87SC114SC154SC340S) were generated (Fig 4C). Substitution of up to four N-terminal cysteines still revealed redox-sensitive *AAGAAT* motif interactions, where variations in band intensities likely reflect different protein levels. However, a complete loss of redox-sensitivity was observed when all five N-terminal cysteine residues were exchanged (PANC27SC68SC87SC114SC154S). Diamide treatment could no longer exert an inhibitory effect on the DNA-binding affinity of this mutagenized PAN protein. The same complete loss of redox-dependent DNA-binding was observed, when all six cysteine residues where mutagenized, showing that Cys340 does likely not exhibit its crucial function via affecting the PAN DNA-binding activity (Fig 4C). These EMSA studies indicate that the redox-dependent PAN DNA-binding is mediated by an interplay between the five N-terminally located cysteines, where Cys154 seems to exhibit the strongest effect on the redox-sensitivity in EMSA studies. However, the mutagenized PANC154S protein can still complement the *pan* mutant [15], supporting the importance of a combined activity of the N-terminal cysteines. The impact of all five N-terminal cysteine residues was further investigated by analyzing a PAN mutant protein where the N-terminus has been removed (PANΔN), leaving only Cys340 intact. As expected, PANΔN protein did not show a reduced DNA-binding under oxidizing conditions (Fig 4D). This observation together with the quintuple cysteine mutant data demonstrates the significance of the PAN N-terminus for mediating a redox-dependent interaction with the regulatory *AAGAAT cis-*element. The in planta activity of the N-terminus was analyzed by expressing *PANΔN* under the control of the *CaMV 35S* promoter in *pan* knockout mutants. Transgenic T_1_ plants (n = 200 flowers, 20 T_1_ plants) failed to complement the *pan* mutant phenotype. Furthermore, mutagenesis of Cys68 and Cys87 as well as altering all five N-terminal cysteines into serines and overexpression in the *pan* mutant could not complement the mutant phenotype (n = 200 flowers, 25 T_1_ plants). This observation strengthens the in vivo importance of the N-terminal cysteines for the PAN function in petal development. The bZIP domain of TGA TFs is localized close to their N-terminus and comprises basic residues such as lysines and arginines that mediate specific interactions with the DNA (Fig 2B) [2]. The close proximity of the N-terminal PAN extension to the bZIP domains may facilitate redox-dependent cysteine modifications that modulate the interaction capacity of the bZIP domain with binding motifs. To summarize, the in vivo analysis shows the importance of the PAN N-terminus for governing flower development and in vitro and in vivo studies reveal the importance of the combined activity of its cysteines to mediate a redox-dependent DNA-binding.

### Evolution of the *AAGAAT* motif

**Fig 5:**
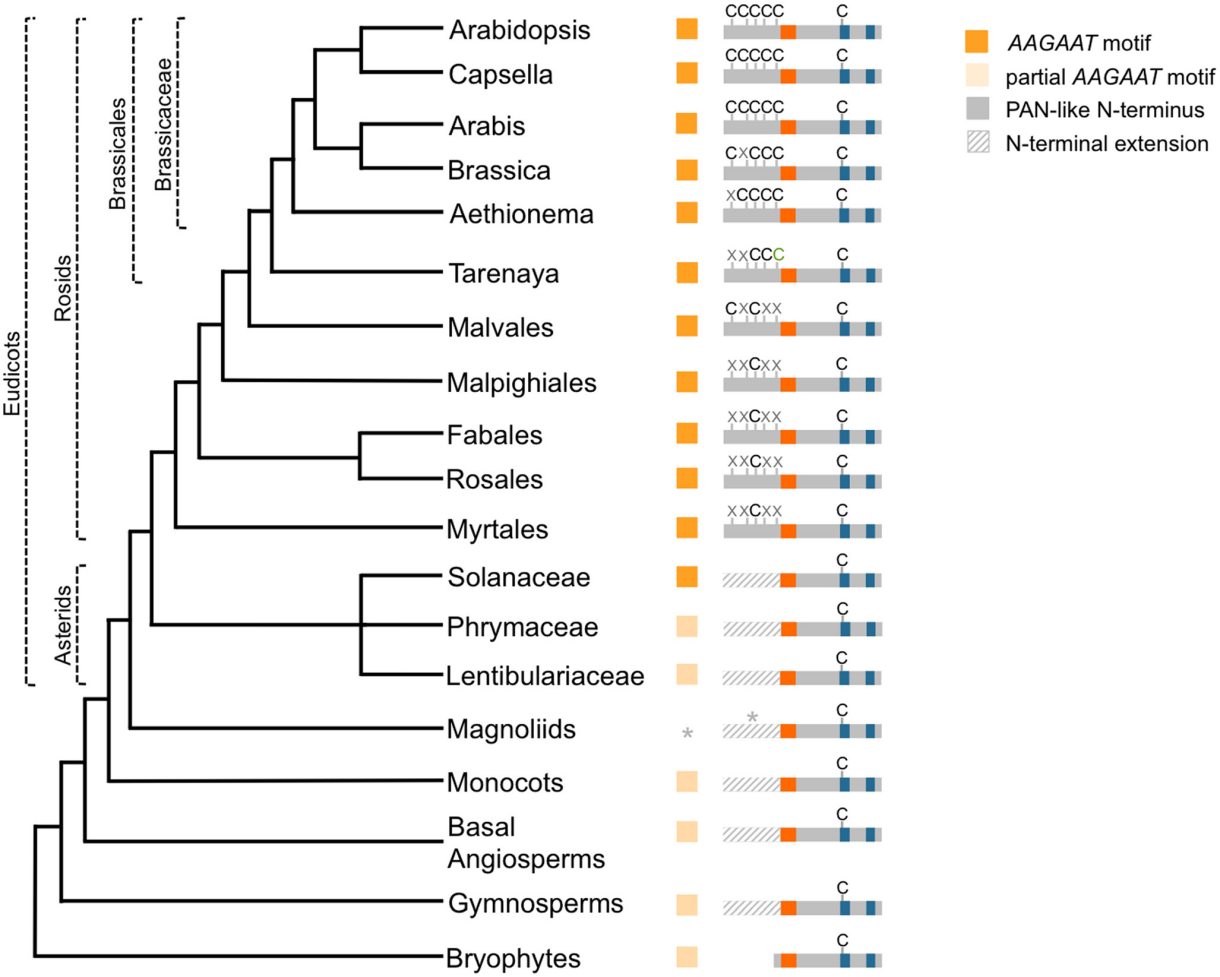
Evolution of the N-terminal PAN extension and the *AAGAAT* motif. Cladogram of evolutionary informative species from seed plants indicating *AAGAAT* motifs and N-terminal extensions of the respective PAN and *AG* homologs. Orange boxes indicate presence of an *AAGAAT* motif comprising the AAGAAT sequence and core TGA binding site. Beige boxes label partial *AAGAAT* motifs, which possess only the AAGAAT sequence and lack the core TGA binding sequence TGACG. Schematic representation of PAN protein homologs shows N-terminal extensions with significant (grey bar) or low sequence homology (striped bar) to the PAN N-terminus or its absence. Presence (C) or absence (X) of cysteines equivalent to the five N-terminal PAN cysteines is depicted. Stars indicate that the definition of a magnoliid PAN homolog start codon was not possible and that no genome sequencing data were available.

The observation that the PAN N-terminus mediates a redox-dependent interaction with the *AAGAAT* motif from the floral TF *AG*, prompted us to analyze the evolutionary relationships between the N-terminus and the *AAGAAT* motif. Earlier studies on conserved elements in the *AG* intronic regions identified *AAGAAT* motifs in diverse angiosperm species [11, 12]. Here, these studies were extended and over 20 species were analyzed from evolutionary informative land plant species for which whole genome DNA sequence information is available, including the gymnosperm *Picea abies* and the bryophyte *Physcomitrella patens*. Fig 5 shows a cladogram of the analyzed taxa, applying the recent grouping of the monocots as a sister clade to the magnoliids and eudicots [27]. The presence of the *AAGAAT* motif is indicated for the investigated species and sequence alignments are shown in S2 Fig. A partial *AAGAAT* motif comprising the AAGAAT sequence but lacking the core TGA TF binding site already exists in the basal-most analyzed land plant, the bryophyte *Physcomitrella patens*. Such partial *AAGAAT* motifs were also identified in representatives of basal angiosperms (*Amborella trichopoda)*, gymnosperms (*Picea abies*) and monocots (*Musa acuminata*, *Oryza sativa*). In asterids, partial motifs exist in the Phrymaceae and Lentibulariaceae and complete *AAGAAT* motifs, where an AAGAAT sequence precedes the TGA binding site, were identified in the Solanaceae. All analyzed rosids comprise a complete *AAGAAT* motif.

### Evolution of the N-terminal PAN extension

Furthermore, presence of the N-terminal PAN cysteines and Cys340 equivalents was investigated in the respective PAN homologs and the alignment is shown in S3 Fig. Whereas Physcomitrella TGA TFs do not possess a N-terminal extension, extensions with a variable length and sequence identity exist in all other investigated PAN homologs from seed plants. However, only in rosids the N-termini of PAN homologs reveal a significant similarity to PAN (S3 Fig). Moreover, only in this land plant group cysteine residues exist in positions equivalent to the PAN cysteines. Interestingly, whereas in Myrtales, Rosales, Fabales, Malpighiales only one and in Malvales two cysteines occur, their number increased exclusively in the Brassicales. In *Tarenaya hassleriana* from the Cleomaceae family, sister to the Brassicaceae, two TGA TFs with high similarity to PAN were identified. One homolog comprises two and the other homolog three N-terminal cysteines (Fig 5 and S3 Fig) and the homologs might have arisen by an ancient genome triplication (Th-α) only experienced by Tarenaya [57]. In the Brassicaceae, five cysteines equivalent to all PAN cysteines exist in the N-terminus of homologs of the core Brassicaceae *Arabidopsis thaliana* and *Capsella rubella* as well as in *Arabis alpina*. In *Brassica napus*, four cysteines were identified and the PANCys68 equivalent is not present. This PAN homolog might be able to exert a PAN-like activity as EMSA studies showed that PAN can still mediate a redox-sensitive DNA-binding when this cysteine has been replaced by a serine (Fig 4B) and Cys68 has also been shown to be dispensable for an in vivo PAN activity [15]. Brassicaceae species probably experienced three ancient whole-genome duplications (WGD) that led to paleopolyploidy, which played an important role in the genetic diversification of this family by providing additional gene copies. The last WGD, At-α, coincides roughly with the radiation of the Brassicaceae, which has likely been driven by extrinsic factors such as climatic changes creating open and drier habitats [58, 59]. The formation of four sepals and four petals in the Brassicaceae flower is exceptional, as the majority of the core eudicots form a pentamerous perianth [60]. The *pan* mutant forms pentamerous outer whorls and it is therefore tempting to speculate that the exclusive evolution of the special PAN N-terminus in the Brassicaceae may have contributed to the special tetramerous perianth morphology. Furthermore, PAN also controls stem cell formation and termination of the floral meristem. This function is revealed when *pan* mutants are cultivated under short-day conditions, where *ag*-like flowers develop with extra organs in the center [9]. As PAN participates in several developmental pathways sensing environmental conditions such as day-length and also other abiotic factors [61], the redox-sensitive N-terminus may be involved in mediating these changes and contributed thereby to the adaptation of the Brassicaceae to novel ecological niches with different climatic conditions. Further PAN studies are required in the future to address the in vivo significance of its cysteines and their participation in redox-processes. For another plant-specific TF, TCP15, it has recently been shown that its activity is redox-modulated by high light intensity and that TCP15 redox-interconversions contribute to a response to altered environmental conditions [35].

### Conclusion

The PAN-like N-terminus with five cysteines is specific for the Brassicaceae, suggesting a specialized function via affecting redox-sensitive DNA-binding in a combinatorial fashion. Contrarily, a PANCys340 equivalent exists in all analyzed land plants suggesting that this cysteine exerts a conserved function in redox-signaling dating back over 450 mya. It has recently been reported that the intracellular distribution of glutathione, a crucial antioxidant, is altered during the cell cycle and that this thiol-tripeptide accumulates in the nucleus in the G1 phase supporting an important function for GSH in nuclear redox-signaling [62–64]. Cys340 is likely not contributing to a redox-sensitive DNA-binding but it can be glutathionylated in vitro and might thereby participate in nuclear redox-signaling processes. Together, these findings emphasizes the impact of different redox-dependent posttranslational modifications in regulating the activity of the plant developmental TF PAN.

## Supporting Information

S1 Fig SDS-PAGE analysis of in vitro produced PAN protein variants using the TnT^™^ SP6-Coupled Reticulocyte Lysate system (A-B). In vitro PAN protein expression was conducted in the presence of radioactive-labeled ^35^S-methionine. 2.5 μl of each reaction mix was analyzed on a 10% SDS gel and protein synthesis was visualized by autoradiography. Full-length PAN protein bands are indicated by triangles and the truncated PANΔN variant by a full circle, asterisks label free unincorporated ^35^S-methionine.
(TIFF)

S2 Fig Alignment of the *AAGAAT* motif from the analyzed *AG*-like intron regions. Comparison of sequences from plant species listed in S1 Table was conducted with the MacVector program. The *AAGAAT* motif from the second Arabidopsis *AG* intron is indicated in bold. The blue box marks the characteristic AAGAAT sequence and the orange box the core TGA TF binding site. The first position of the *AAGAAT* motif is variable [11]. The *Aethionema arabicum* motif originated from a BLAST result, indicated by a star symbol. The *Oryza sativa AAGAAT* motif (circle) was obtained from [12]. At *Arabidopsis thaliana*; Al *Arabidopsis lyrata*; Cr *Capsella rubella*; Ag *Arabis gunnisoniana*; Br *Brassica rapa*; Bo *Brassica oleracea;* Aa *Aethionema arabicum*; Th *Tarenaya hasseleriana*; Tc *Theobroma cacao;* Gr *Gossypium raimondii*; Pt *Populus trichocarpa*; Gm *Glycine max*; Md *Malus domestica*; Fv *Fragaria vesca*; Pp *Prunus persica*; Eg *Eucalyptus grandis*; St *Solanum tuberosum*; Mg *Mimulus guttatus*; Ug *Utricularia gibba*; Ma *Musa acuminata*; Os *Oryza sativa*; Atr *Amborella trichopoda*; Pa *Picea abies*; Ppa *Physcomitrella patens*.
(TIF)

S3 Fig Sequence alignment of the analyzed PAN homologs. Amino acid sequences from the plant species listed in the S1 Table were compared using the CLUSTALW program and adjusted manually. The *Arabidopsis thaliana* PAN sequence is indicated in bold. Cysteines at positions equivalent to the PAN cysteines are highlighted in red. At *Arabidopsis thaliana*; Al *Arabidopsis lyrata*; Cr *Capsella rubella*; Aal *Arabis alpina*; Br *Brassica rapa*; Bo *Brassica oleracea;* Aa *Aethionema arabicum*; Th *Tarenaya hasseleriana*; Tc *Theobroma cacao;* Gr *Gossypium raimondii*; Pt *Populus trichocarpa*; Gm *Glycine max*; Md *Malus domestica*; Fv *Fragaria vesca*; Pp *Prunus persica*; Eg *Eucalyptus grandis*; St *Solanum tuberosum*; Mg *Mimulus guttatus*; Ug *Utricularia gibba*; Ma *Musa acuminata*; Os *Oryza sativa*; Atr *Amborella trichopoda*; Pa *Picea abies*; Ppa *Physcomitrella patens*.
(TIF)

S1 Table Oligonucleotides used in this study (A) accession numbers and sources of analyzed genes and proteins (B-C). (PDF)

## References

[1] Running MP, Meyerowitz EM: Mutations in the PERIANTHIA gene of Arabidopsis specifically alter floral organ number and initiation pattern. *Development* 1996, 122(4):1261–1269. 8620853 10.1242/dev.122.4.1261

[2] Gatz C: From pioneers to team players: TGA transcription factors provide a molecular link between different stress pathways. *Mol Plant Microbe Interact* 2013, 26(2):151–159. 10.1094/MPMI-04-12-0078-IA 23013435

[3] Lam E, Chua NH: Asf-2—a Factor That Binds to the Cauliflower Mosaic Virus-35s Promoter and a Conserved Gata Motif in Cab Promoters. *Plant Cell* 1989, 1(12):1147–1156. 2535536 10.1105/tpc.1.12.1147 PMC159850

[4] Lebel E, Heifetz P, Thorne L, Uknes S, Ryals J, Ward E: Functional analysis of regulatory sequences controlling PR-1 gene expression in Arabidopsis. *Plant J* 1998, 16(2):223–233. 9839467 10.1046/j.1365-313x.1998.00288.x

[5] Garreton V, Carpinelli J, Jordana X, Holuigue L: The as-1 promoter element is an oxidative stress-responsive element and salicylic acid activates it via oxidative species. *Plant Physiology* 2002, 130(3):1516–1526. 12428016 10.1104/pp.009886 PMC166670

[6] Xiang C, Miao Z, Lam E: DNA-binding properties, genomic organization and expression pattern of TGA6, a new member of the TGA family of bZIP transcription factors in Arabidopsis thaliana. *Plant Mol Biol* 1997, 34(3):403–415. 9225852 10.1023/a:1005873500238

[7] Johnson C, Glover G, Arias J: Regulation of DNA binding and trans-activation by a xenobiotic stress-activated plant transcription factor. *Journal of Biological Chemistry* 2001, 276(1):172–178. 11034999 10.1074/jbc.M005143200

[8] Chen WQ, Singh KB: The auxin, hydrogen peroxide and salicylic acid induced expression of the Arabidopsis GST6 promoter is mediated in part by an ocs element. *Plant Journal* 1999, 19(6):667–677. 10571852 10.1046/j.1365-313x.1999.00560.x

[9] Maier AT, Stehling-Sun S, Wollmann H, Demar M, Hong RL, Haubeiss S et al: Dual roles of the bZIP transcription factor PERIANTHIA in the control of floral architecture and homeotic gene expression. *Development* 2009, 136(10):1613–1620. 10.1242/dev.033647 19395639 PMC2673762

[10] Das P, Ito T, Wellmer F, Vernoux T, Dedieu A, Traas J et al: Floral stem cell termination involves the direct regulation of AGAMOUS by PERIANTHIA. *Development* 2009, 136(10):1605–1611. 10.1242/dev.035436 19395638

[11] Hong RL, Hamaguchi L, Busch MA, Weigel D: Regulatory elements of the floral homeotic gene AGAMOUS identified by phylogenetic footprinting and shadowing. *Plant Cell* 2003, 15(6):1296–1309. 12782724 10.1105/tpc.009548 PMC156367

[12] Causier B, Bradley D, Cook H, Davies B: Conserved intragenic elements were critical for the evolution of the floral C-function. *Plant Journal* 2009, 58(1):41–52. 10.1111/j.1365-313X.2008.03759.x 19054363

[13] Coen ES, Meyerowitz EM: The War of the Whorls—Genetic Interactions Controlling Flower Development. *Nature* 1991, 353(6339):31–37. 1715520 10.1038/353031a0

[14] Sieburth LE, Running MP, Meyerowitz EM: Genetic separation of third and fourth whorl functions of AGAMOUS. *Plant Cell* 1995, 7(8):1249–1258. 7549481 10.1105/tpc.7.8.1249 PMC160948

[15] Li S, Lauri A, Ziemann M, Busch A, Bhave M, Zachgo S: Nuclear activity of ROXY1, a glutaredoxin interacting with TGA factors, is required for petal development in Arabidopsis thaliana. *Plant Cell* 2009, 21(2):429–441. 10.1105/tpc.108.064477 19218396 PMC2660636

[16] Gutsche N, Thurow C, Zachgo S, Gatz C: Plant-specific CC-type glutaredoxins: functions in developmental processes and stress responses. *Biological Chemistry* 2015, 396(5):495–509. 10.1515/hsz-2014-0300 25781542

[17] Murmu J, Bush MJ, DeLong C, Li S, Xu M, Khan M et al: Arabidopsis basic leucine-zipper transcription factors TGA9 and TGA10 interact with floral glutaredoxins ROXY1 and ROXY2 and are redundantly required for anther development. *Plant Physiol* 2010, 154(3):1492–1504. 10.1104/pp.110.159111 20805327 PMC2971623

[18] Kesarwani M, Yoo J, Dong X: Genetic interactions of TGA transcription factors in the regulation of pathogenesis-related genes and disease resistance in Arabidopsis. *Plant Physiol* 2007, 144(1):336–346. 17369431 10.1104/pp.106.095299 PMC1913812

[19] Despres C, Chubak C, Rochon A, Clark R, Bethune T, Desveaux D et al: The Arabidopsis NPR1 disease resistance protein is a novel cofactor that confers redox regulation of DNA binding activity to the basic domain/leucine zipper transcription factor TGA1. *Plant Cell* 2003, 15(9):2181–2191. 12953119 10.1105/tpc.012849 PMC181339

[20] Verelst W, Saedler H, Muenster T: MIKC* MADS-protein complexes bind motifs enriched in the proximal region of late pollen-specific Arabidopsis promoters. *Plant Physiology* 2007, 143(1):447–460. 17071640 10.1104/pp.106.089805 PMC1761959

[21] Cole SR, Ma X, Zhang X, Xia Y: Electron transfer dissociation (ETD) of peptides containing intrachain disulfide bonds. *J Am Soc Mass Spectrom* 2012, 23(2):310–320. 10.1007/s13361-011-0300-z 22161508

[22] Perkins DN, Pappin DJC, Creasy DM, Cottrell JS: Probability-based protein identification by searching sequence databases using mass spectrometry data. *Electrophoresis* 1999, 20(18):3551–3567. 10612281 10.1002/(SICI)1522-2683(19991201)20:18<3551::AID-ELPS3551>3.0.CO;2-2

[23] Proost S, Van Bel M, Vaneechoutte D, Van de Peer Y, Inze D, Mueller-Roeber B et al: PLAZA 3.0: an access point for plant comparative genomics. *Nucleic Acids Research* 2015, 43(D1):D974–D981. 25324309 10.1093/nar/gku986 PMC4384038

[24] Goodstein DM, Shu S, Howson R, Neupane R, Hayes RD, Fazo J et al: Phytozome: a comparative platform for green plant genomics. *Nucleic Acids Res* 2012, 40(Database issue):D1178–1186. 10.1093/nar/gkr944 22110026 PMC3245001

[25] Sundell D, Mannapperuma C, Netotea S, Delhomme N, Lin YC, Sjodin A et al: The Plant Genome Integrative Explorer Resource: PlantGenIE.org. *The New phytologist* 2015, 208(4):1149–1156. 10.1111/nph.13557 26192091

[26] Lyons E, Pedersen B, Kane J, Alam M, Ming R, Tang HB et al: Finding and Comparing Syntenic Regions among Arabidopsis and the Outgroups Papaya, Poplar, and Grape: CoGe with Rosids. *Plant Physiology* 2008, 148(4):1772–1781. 10.1104/pp.108.124867 18952863 PMC2593677

[27] Zeng LP, Zhang Q, Sun RR, Kong HZ, Zhang N, Ma H: Resolution of deep angiosperm phylogeny using conserved nuclear genes and estimates of early divergence times. *Nature communications* 2014, 5. 10.1038/ncomms5956 PMC4200517 25249442

[28] Pape S, Thurow C, Gatz C: The Arabidopsis PR-1 promoter contains multiple integration sites for the coactivator NPR1 and the repressor SNI1. *Plant Physiol* 2010, 154(4):1805–1818. 10.1104/pp.110.165563 20935179 PMC2996008

[29] Fang Q, Chen S, Wang Y, Jiang S, Zhang R, Hu C et al: Functional analyses of the mutation nt-128 T -> G in the hepatocyte nuclear factor-1 alpha promoter region in Chinese diabetes pedigrees. *Diabetic Med* 2012, 29(11):1456–1464. 10.1111/j.1464-5491.2012.03626.x 22413961 PMC3570122

[30] Seldeen KL, McDonald CB, Deegan BJ, Farooq A: Single nucleotide variants of the TGACTCA motif modulate energetics and orientation of binding of the Jun-Fos heterodimeric transcription factor. *Biochemistry* 2009, 48(9):1975–1983. 10.1021/bi802068s 19215067 PMC2693225

[31] Shaikhali J, Heiber I, Seidel T, Stroher E, Hiltscher H, Birkmann S et al: The redox-sensitive transcription factor Rap2.4a controls nuclear expression of 2-Cys peroxiredoxin A and other chloroplast antioxidant enzymes. *BMC Plant Biol* 2008, 8:48 10.1186/1471-2229-8-48 18439303 PMC2386467

[32] Shaikhali J, Noren L, de Dios Barajas-Lopez J, Srivastava V, Konig J, Sauer UH et al: Redox-mediated mechanisms regulate DNA binding activity of the G-group of basic region leucine zipper (bZIP) transcription factors in Arabidopsis. *J Biol Chem* 2012, 287(33):27510–27525. 10.1074/jbc.M112.361394 22718771 PMC3431687

[33] Tron AE, Bertoncini CW, Chan RL, Gonzalez DH: Redox regulation of plant homeodomain transcription factors. *J Biol Chem* 2002, 277(38):34800–34807. 12093803 10.1074/jbc.M203297200

[34] Viola IL, Guttlein LN, Gonzalez DH: Redox modulation of plant developmental regulators from the class I TCP transcription factor family. *Plant Physiol* 2013, 162(3):1434–1447. 10.1104/pp.113.216416 23686421 PMC3707549

[35] Viola IL, Camoirano A, Gonzalez DH: Redox-Dependent Modulation of Anthocyanin Biosynthesis by the TCP Transcription Factor TCP15 during Exposure to High Light Intensity Conditions in Arabidopsis. *Plant Physiol* 2016, 170(1):74–85. 10.1104/pp.15.01016 26574599 PMC4704573

[36] Choi J, Huh SU, Kojima M, Sakakibara H, Paek KH, Hwang I: The cytokinin-activated transcription factor ARR2 promotes plant immunity via TGA3/NPR1-dependent salicylic acid signaling in Arabidopsis. *Dev Cell* 2010, 19(2):284–295. 10.1016/j.devcel.2010.07.011 20708590

[37] Farinati S, DalCorso G, Varotto S, Furini A: The Brassica juncea BjCdR15, an ortholog of Arabidopsis TGA3, is a regulator of cadmium uptake, transport and accumulation in shoots and confers cadmium tolerance in transgenic plants. *The New phytologist* 2010, 185(4):964–978. 10.1111/j.1469-8137.2009.03132.x 20028476

[38] Foley RC, Singh KB: TGA5 acts as a positive and TGA4 acts as a negative regulator of ocs element activity in Arabidopsis roots in response to defence signals. *Febs Letters* 2004, 563(1–3):141–145. 15063738 10.1016/S0014-5793(04)00288-1

[39] Shearer HL, Cheng YT, Wang L, Liu J, Boyle P, Despres C et al: Arabidopsis clade I TGA transcription factors regulate plant defenses in an NPR1-independent fashion. *Mol Plant Microbe Interact* 2012, 25(11):1459–1468. 10.1094/MPMI-09-11-0256 22876961

[40] Zhang Y, Tessaro MJ, Lassner M, Li X: Knockout analysis of Arabidopsis transcription factors TGA2, TGA5, and TGA6 reveals their redundant and essential roles in systemic acquired resistance. *Plant Cell* 2003, 15(11):2647–2653. 14576289 10.1105/tpc.014894 PMC280568

[41] Zander M, La Camera S, Lamotte O, Metraux JP, Gatz C: Arabidopsis thaliana class-II TGA transcription factors are essential activators of jasmonic acid/ethylene-induced defense responses. *Plant J* 2010, 61(2):200–210. 10.1111/j.1365-313X.2009.04044.x 19832945

[42] Fan WH, Dong XN: In vivo interaction between NPR1 and transcription factor TGA2 leads to salicylic acid-mediated gene activation in Arabidopsis. *Plant Cell* 2002, 14(6):1377–1389. 12084833 10.1105/tpc.001628 PMC150786

[43] Durrant WE, Dong X: Systemic acquired resistance. *Annual review of phytopathology* 2004, 42:185–209. 15283665 10.1146/annurev.phyto.42.040803.140421

[44] Zander M, La Camera S, Lamotte O, Metraux JP, Gatz C: Arabidopsis thaliana class-II TGA transcription factors are essential activators of jasmonic acid/ethylene-induced defense responses. *Plant Journal* 2010, 61(2):200–210. 10.1111/j.1365-313X.2009.04044.x 19832945

[45] Lindermayr C, Sell S, Muller B, Leister D, Durner J: Redox regulation of the NPR1-TGA1 system of Arabidopsis thaliana by nitric oxide. *Plant Cell* 2010, 22(8):2894–2907. 10.1105/tpc.109.066464 20716698 PMC2947166

[46] Wiesner J, Premsler T, Sickmann A: Application of electron transfer dissociation (ETD) for the analysis of posttranslational modifications. *Proteomics* 2008, 8(21):4466–4483. 10.1002/pmic.200800329 18972526

[47] Wu SL, Jiang HT, Hancock WS, Karger BL: Identification of the Unpaired Cysteine Status and Complete Mapping of the 17 Disulfides of Recombinant Tissue Plasminogen Activator Using LC-MS with Electron Transfer Dissociation/Collision Induced Dissociation. *Anal Chem* 2010, 82(12):5296–5303. 10.1021/ac100766r 20481521 PMC2890214

[48] Cao X, Kambe F, Lu XL, Kobayashi N, Ohmori S, Seo H: Glutathionylation of two cysteine residues in paired domain regulates DNA binding activity of Pax-8. *Journal of Biological Chemistry* 2005, 280(27):25901–25906. 15888455 10.1074/jbc.M411443200

[49] Klatt P, Molina EP, Lamas S: Nitric oxide inhibits c-Jun DNA binding by specifically targeted S-glutathionylation. *Journal of Biological Chemistry* 1999, 274(22):15857–15864. 10336489 10.1074/jbc.274.22.15857

[50] Pineda-Molina E, Lamas S: S-glutathionylation of NF-kappa B subunit p50. *Methods Enzymol* 2002, 359:268–279. 12481579 10.1016/s0076-6879(02)59191-6

[51] Qanungo S, Starke DW, Pai HV, Mieyal JJ, Nieminen AL: Glutathione supplementation potentiates hypoxic apoptosis by S-glutathionylation of p65-NFkappaB. *J Biol Chem* 2007, 282(25):18427–18436. 17468103 10.1074/jbc.M610934200

[52] Liao BC, Hsieh CW, Lin YC, Wung BS: The glutaredoxin/glutathione system modulates NF-kappaB activity by glutathionylation of p65 in cinnamaldehyde-treated endothelial cells. *Toxicol Sci* 2010, 116(1):151–163. 10.1093/toxsci/kfq098 20351055

[53] Klatt P, Lamas S: Regulation of protein function by S-glutathiolation in response to oxidative and nitrosative stress. *European journal of biochemistry / FEBS* 2000, 267(16):4928–4944. 10931175 10.1046/j.1432-1327.2000.01601.x

[54] Chi YH, Paeng SK, Kim MJ, Hwang GY, Melencion SM, Oh HT et al: Redox-dependent functional switching of plant proteins accompanying with their structural changes. *Front Plant Sci* 2013, 4:277 10.3389/fpls.2013.00277 23898340 PMC3724125

[55] Delaunay A, Pflieger D, Barrault MB, Vinh J, Toledano MB: A thiol peroxidase is an H2O2 receptor and redox-transducer in gene activation. *Cell* 2002, 111(4):471–481. 12437921 10.1016/s0092-8674(02)01048-6

[56] Heine GF, Hernandez JM, Grotewold E: Two cysteines in plant R2R3 MYB domains participate in REDOX-dependent DNA binding. *J Biol Chem* 2004, 279(36):37878–37885. 15237103 10.1074/jbc.M405166200

[57] Schranz ME, Mitchell-Olds T: Independent ancient polyploidy events in the sister families Brassicaceae and Cleomaceae. *Plant Cell* 2006, 18(5):1152–1165. 16617098 10.1105/tpc.106.041111 PMC1456871

[58] Couvreur TLP, Franzke A, Al-Shehbaz IA, Bakker FT, Koch MA, Mummenhoff K: Molecular Phylogenetics, Temporal Diversification, and Principles of Evolution in the Mustard Family (Brassicaceae). *Mol Biol Evol* 2010, 27(1):55–71. 10.1093/molbev/msp202 19744998

[59] Franzke A, German D, Al-Shehbaz IA, Mummenhoff K: Arabidopsis family ties: molecular phylogeny and age estimates in Brassicaceae. *Taxon* 2009, 58(2):425–437.

[60] Soltis DE, Senters AE, Zanis MJ, Kim S, Thompson JD, Soltis PS et al: Gunnerales are sister to other core eudicots: Implications for the evolution of pentamery. *Am J Bot* 2003, 90(3):461–470. 10.3732/ajb.90.3.461 21659139

[61] Maier AT, Stehling-Sun S, Offenburger SL, Lohmann JU: The bZIP Transcription Factor PERIANTHIA: A Multifunctional Hub for Meristem Control. *Front Plant Sci* 2011, 2:79 10.3389/fpls.2011.00079 22645551 PMC3355747

[62] Vivancos PD, Dong Y, Ziegler K, Markovic J, Pallardo FV, Pellny TK et al: Recruitment of glutathione into the nucleus during cell proliferation adjusts whole-cell redox homeostasis in Arabidopsis thaliana and lowers the oxidative defence shield. *Plant J* 2010, 64(5):825–838. 10.1111/j.1365-313X.2010.04371.x 21105929

[63] Markovic J, Borras C, Ortega A, Sastre J, Vina J, Pallardo FV: Glutathione is recruited into the nucleus in early phases of cell proliferation. *Journal of Biological Chemistry* 2007, 282(28):20416–20424. 17452333 10.1074/jbc.M609582200

[64] Vivancos PD, Wolff T, Markovic J, Pallardo FV, Foyer CH: A nuclear glutathione cycle within the cell cycle. *Biochemical Journal* 2010, 431:169–178. 10.1042/BJ20100409 20874710

