# Supplementary figures and images for: The N-Terminus of the Floral Arabidopsis TGA Transcription Factor PERIANTHIA Mediates Redox-Sensitive DNA-Binding
Source: PLoS One. 2016 Apr 29;11(4):e0153810. doi: 10.1371/journal.pone.0153810 (PMC4851370; doi:10.1371/journal.pone.0153810)

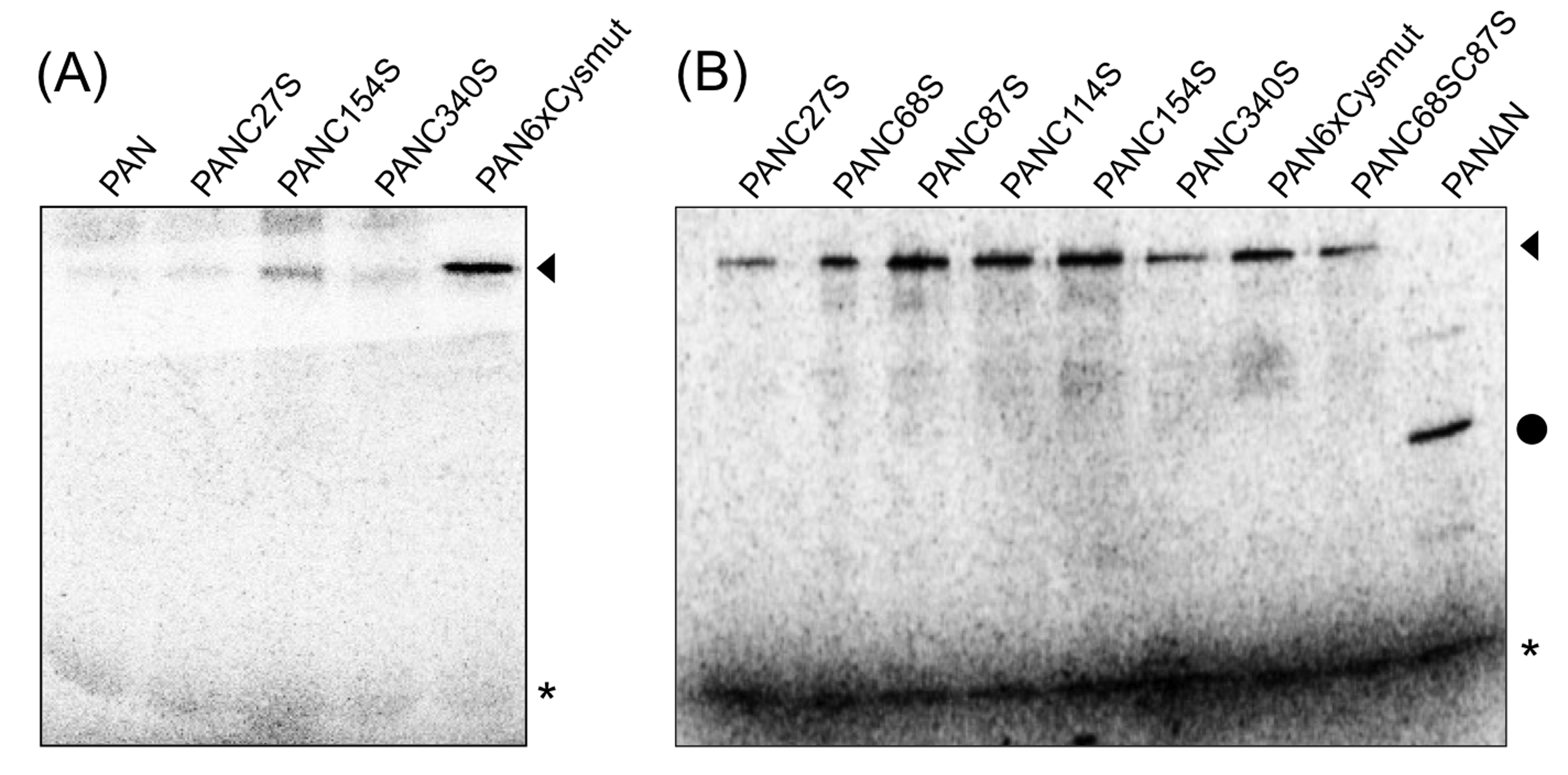

Supplement: S1 Fig — In vitro PAN protein expression was conducted in the presence of radioactive-labeled 35S-methionine. 2.5 μl of each reaction mix was analyzed on a 10% SDS gel and protein synthesis was visualized by autoradiography. Full-length PAN protein bands are indicated by triangles and the truncated PANΔN variant by a full circle, asterisks label free unincorporated 35S-methionine. (TIFF) [file pone.0153810.s001.tiff]

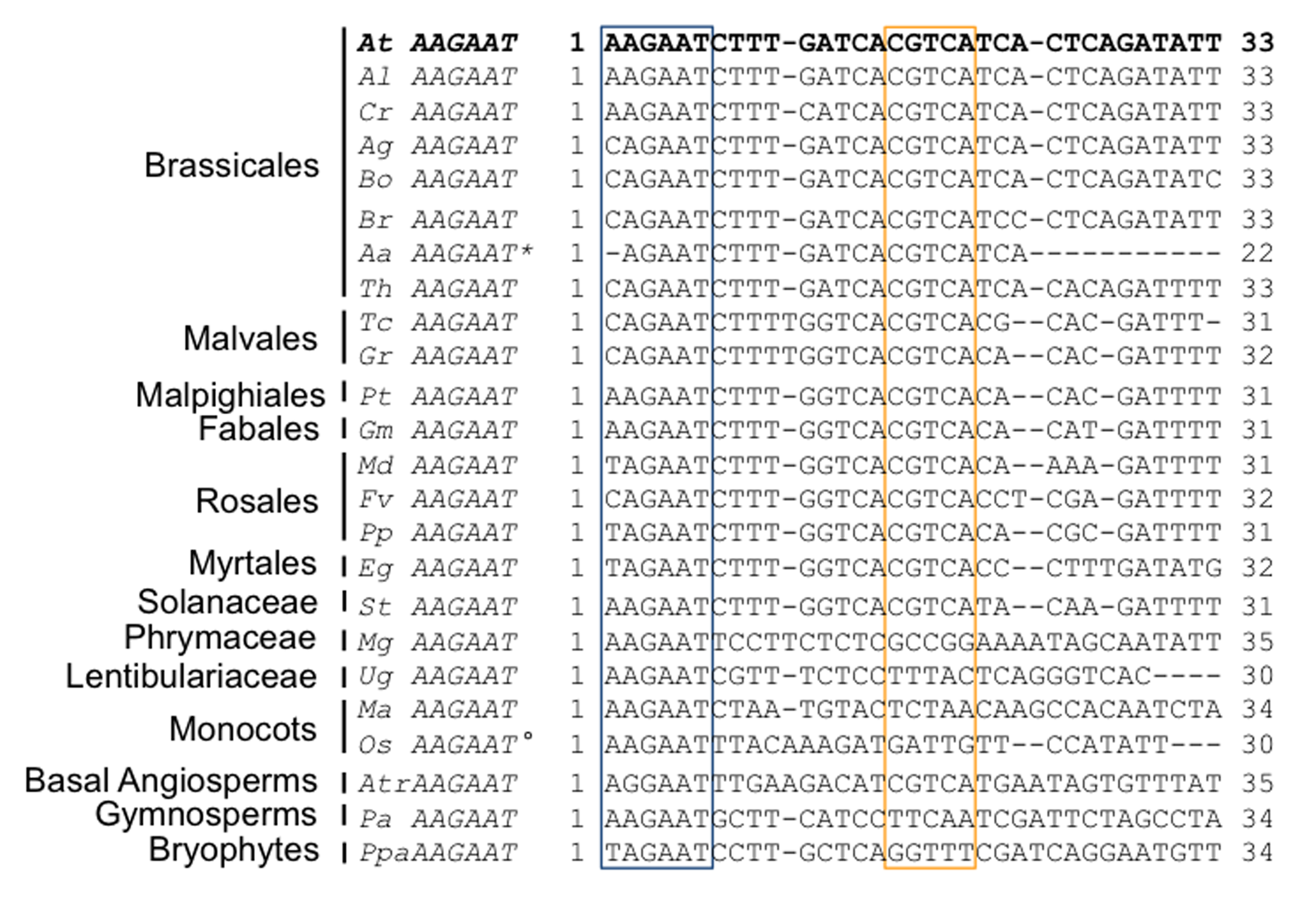

Supplement: S2 Fig — Comparison of sequences from plant species listed in S1 Table was conducted with the MacVector program. The AAGAAT motif from the second Arabidopsis AG intron is indicated in bold. The blue box marks the characteristic AAGAAT sequence and the orange box the core TGA TF binding site. The first position of the AAGAAT motif is variable [11]. The Aethionema arabicum motif originated from a BLAST result, indicated by a star symbol. The Oryza sativa AAGAAT motif (circle) was obtained from [12]. At Arabidopsis thaliana; Al Arabidopsis lyrata; Cr Capsella rubella; Ag Arabis gunnisoniana; Br Brassica rapa; Bo Brassica oleracea; Aa Aethionema arabicum; Th Tarenaya hasseleriana; Tc Theobroma cacao; Gr Gossypium raimondii; Pt Populus trichocarpa; Gm Glycine max; Md Malus domestica; Fv Fragaria vesca; Pp Prunus persica; Eg Eucalyptus grandis; St Solanum tuberosum; Mg Mimulus guttatus; Ug Utricularia gibba; Ma Musa acuminata; Os Oryza sativa; Atr Amborella trichopoda; Pa Picea abies; Ppa Physcomitrella patens. (TIF) [file pone.0153810.s002.tif]
