# Supplementary material for: The N-Terminus of the Floral Arabidopsis TGA Transcription Factor PERIANTHIA Mediates Redox-Sensitive DNA-Binding
Source: PLoS One. 2016 Apr 29;11(4):e0153810. doi: 10.1371/journal.pone.0153810 (PMC4851370; doi:10.1371/journal.pone.0153810)
Supplement: S1 Table — (PDF) [file pone.0153810.s004.pdf]

S1 Table. Oligonucleotides used in this study (A) and the accession numbers as well as the source of the analysed genes and proteins (B-C).

A)

**Name**	**Sequence**
PAN pMAL Fwd *Xmn*I	TGCTCGGAAGGATTTCAATGCAGAGC AGC
PAN pMAL Rev *Nco*I	TACTCAGGCCATGGTTAGTCTCTAGGT
PAN pBAR35S Fwd X*mnI*	GCGCCCGGGATGCAGAGCAGCTTCAA A
PANΔN pBAR35S Fwd *Xma*I	GCGCCCGGGATGACACTTCGTCGACTT
PAN pBAR35S Rev *Xba*I	TCTAGATTAGTCTCTAGGTCTGGCTAA
PAN pTNT Fwd *Kpn*I	CGGGGTACCATGCAGAGCAGCTTCA
PAN pTNT Rev *Xba*I	GCTCTAGATTAGTCTCTAGGTCTGG
PANΔN pTNT Fwd *Kpn*I	CGCGGTACCATGACACTTCGTCGACTT
PAN6xCysmut Rev 1 (template PANC27S)	ACCACCAGAATCCATAACCGTATCAAA
PAN6xCysmut Fwd 1 (PANC154S)	TTTGATACGGTTATGGATTCTGGTGGT GGTGGTGGTGGTGGCTTGAGGGAGA GACTTGAAGGAGGAGAAGAGGAGTCT TTG
PAN6xCysmut Rev 2 (PANC154S)	CCTGAATATTTCATCATAGTGACTCATC ACAGCATCCACTA
PAN6xCysmut Fwd 2 (PANC340S)	ATCGGGTGTGAATTCGCAGTTAGGTG
PAN6xCysmut Fwd 3 (PANC27SC68SC87SC114S340S)	GTGAACAGCAGTTGGTCTGATTCG
PAN6xCysmut Rev 3 (PANC27SC68SC87SC114S340S)	TGAAACCGAATCAGACCAACTGCT
TGA1 pTNT Fwd *Spe*I	GCGACTAGTATGAATTCGACATCGAC
TGA1 pTNT Rev *Xho*I	GCGCTCGAGCGTTGGTTCACGATGTCGAG
TGA2 pTNT Fwd *Kpn*I	CGGTACCATGGCTGATACCAGTCCG
TGA2 pTNT Rev *Xba*I	GCTCTAGATCACTCTCTGGGTCGAG
TGA3 pTNT Fwd *Kpn*I	CGGTACCATGGAGATGATGAGCTCT
TGA3 pTNT Rev *Xba*I	CGTCTAGATCAAGTGTGTTCTCGTGGA
TGA10 pTNT Fwd *Kpn*I	CGGGTACCATGCAAGGTCATCACCA
TGA10 pTNT Rev *Xba*I	CCGTCTAGATTATCCATCTTGCCGAGG
as-1-like Fwd	CTACGTCACTATTTTAC**T**TACGTCATAG
as-1-like Rev	CTATGACGTAAGTAAAATAGTGACGTAG
AAGAAT Fwd	AAGAATCTTTGATCACGTCATCACTCAGATATT
AAGAAT Rev	AATATCTGAGTGATGACGTGATCAAAGATTCTT
ΔbZIP Fwd	AAGAATCTTTGATCAAATCATCACTCAGATATT
ΔbZIP Rev	AATATCTGAGTGATGATTTGATCAAAGATTCTT

B)

Gene	Accession	Source
TGA1	At5g65210	TAIR
TGA3	At1g22070	TAIR
TGA10	At5g06830.3	TAIR
PR1	At2g14610	TAIR

C)

Organism	PAN-like accession	Source	*AG*-like accession	Source
Arabidopsis thaliana	At1g68640	TAIR	At4g18960	TAIR
Arabidopsis lyrata	XP_002888675	NCBI	Al7g23670	PLAZA
Capsella rubella	XP_006300957	NCBI	scaffold7	BRAD
Arabis	KFK41287 (*A. alpina*)	NCBI	AY253244 (*A. gunnisoniana*)	NCBI
Brassica oleracea	XP_013590283	NCBI	AY253241	NCBI
Brassica rapa	XP_009105416	NCBI	Br01g10090	PLAZA
Aethionema arabicum	scaffold 6481_3	BRAD	scaffold 2391	BRAD
Tarenaya hassleriana	XP_010551287; XP_010532205	NCBI	Th2v27289 XM_010520563	CoGe NCBI
Theobroma cacao	XP_007046280	NCBI	Tc0006g15800	PLAZA
Gossypium raimondii	XP_012438631	NCBI	Gr00g01610	PLAZA
Populus trichocarpa	XP_002311449	NCBI	Pt11g07580	PLAZA
Glycine max	XP_006574877	NCBI	Gm13g29510	PLAZA
Fragaria vesca	XP_011462928	NCBI	Fv3g08310	PLAZA
Malus domestica	XP_008339696	NCBI	Md10g021630	PLAZA
Prunus perisca	XP_007226716	NCBI	Ppe004g06700	PLAZA
Eucalyptus grandis	XP_010066737	NCBI	Eg0005g21900	PLAZA
Solanum tuberosum	XP_006362551	NCBI	St02g018200	PLAZA
Mimulus guttatus	Migut.L01088.1	phytozome	MigutC01334	phytozome
Utricularia gibba	Scf000t50.g5464.t1	CoGe	scf00662.g6304	CoGe
Liriodendron tulipifera	b4_c11119	AAGP	-	-
Musa acuminata	XP_009417650	NCBI	GSMuA_Achr10g10990_001	phytozome
Orytza sativa	Os05g37170	PLAZA	OsMADS3	Causier et al. 2009
Pinus abies	MA_130907g0010	congenie	MA_20571	congenie
Physcomitrella patens	Pp00126g01020	PLAZA	Pp012g080000	phytozome
